# Popular Hygienic and Medical Lectures

**Published:** 1849-05

**Authors:** 


*Popular Hygienic and Medical Lectures.*—For the past eight or ten weeks, our city has been visited by one of the numerous fraternity of self-styled "Professors"—save the mark! In these latter days of plenitude of diluted knowledge, we have such Professors made by imbibition from the Pierian spring, as a sponge absorbs water. This prefix is unfurled by almost every rover in the high seas of science, art, or even mechanical occupation, and they thrive by it; therein lies the secret; driving a flourishing business, by preying upon the full-freighted credulity, or curiosity of a portion of community, mentally endowed and fitted to their designs.

As this "Professor" has had a more than usual success in his slight of hand practices here, we are tempted to step aside from our prescribed course, and exhibit some of the mysteries latent, now unfolded by him. The almost defunct *science* of Mesmerism, as used for medical purposes, has been galvanized into new being, to suit his purpose, and now we are informed by him, that the galvanic and the mesmeric agent are identical! His want of success in procuring the requisite number of subjects for an evening's exhibit, is apologised for, by account of the extreme heaviness of the air, and this on a rainy day!—with sundry other *new facts*, set forth in what is called a series of lectures, defying the king's English, and withal, so disjointed, as not to contain enough of dissevered members to admit of dissection by the veriest tyro of a school-boy, read in Lindley Murray, or besmattered in any science, by him referred to. By vote of his audience, so says drill sergeant puffer, in the daily papers, he prolongs this fare to the eighth course, by unanimous vote of his audience. Yet night after night, his audience has been numerous, and, as a whole, respectably attended! The usual newspaper puffs have been proffered liberally, and scarcely exceeded by the effrontery of his own announcement. "By the unanimous request of the Young Men's Association," whose rooms he occupies.

That the Young Men's Association will realize quite a harvest, to add to their scanty funds, in use of their name, unasked, gleaned by lease of rooms for such purposes, is all the good we can discover from this not only high-handed effrontery, but crying ill of the times—a contraband exhibit of spurious wares, misnamed scientific lectures, on medical topics, nominally by a titled Professor.

Similar in design, if not in character, are the lectures periodically given by a so-called Dr. Whiton, of whom we know but little, but presume, from his practice, to be the itinerant vender, acting as agent for some one of the many manufacturers of patent shoulder-braces. We need not, to the

[illegible] [illegible]an, say, of no earthly application can shoulder-braces be, as a re[illegible]dial agent for phthisis tuberculosis, for which they are commended. Ma[illegible] have been first duped to believe they were afflicted with the disease, and then induced to wear them, thus suffering mentally, physically, and finally, to the extreme of their pecuniary ability.

As we remarked of the "Professor," and he is but one of a genus, (so is it true of this lecturer,) he succeeds in commanding marvelously large audiences—audiences far out-numbering usual attendants upon those lectures, the subjects of which are selected with greatest care, by men known for their attainments, and constituting a literary and scientific course before the Young Men's Association, each winter season—a season of greatest leisure for lecture-going. We ask, why is this? Is there any peculiar pleasure in listening to a rehearsal of the physical ills of life, until wrought into excited belief of their self-verity? If there is, let these newly-infected Professors pander to the itch of such desire. But, if on the contrary, there is really, not a morbid, but [illegible] desire for knowledge of the laws governing our physical bein[illegible] to the end that life may be cheered, preserved and prolonged, coupled with a rational love of learning, and this no idle curiosity, we would suggest that the Lecture Committee of the Young Men's Association, in their official capacity, first procure and then announce such a series as will meet that demand, the forth-coming lecture season.

